# Pulmonary arteriovenous malformation and follow-up imaging

**DOI:** 10.11604/pamj.2020.37.294.27100

**Published:** 2020-12-02

**Authors:** Pahnwat Tonya Taweesedt, Salim Surani

**Affiliations:** 1 Department of Pulmonary Medicine, Corpus Christi Medical Center, Texas, United States of America,; 2 Texas A & M University, Texas, United States of America,; 3 Pulmonary Medicine Fellowship Program, Corpus Christi Medical Center, Texas, United States of America

**Keywords:** Pulmonary arteriovenous malformation, Osler-Weber-Rendu syndrome, hereditary hemorrhagic telangiectasia

## Image in medicine

An 85-year-old Caucasian female with past medical history of hypertension, hyperlipidemia, polymyalgia rheumatica, coronary artery disease, Osler-Weber-Rendu syndrome (diagnosed 18 years ago), intermittent epistaxis and pulmonary arteriovenous malformation (AVM) was referred to the clinic for abnormal chest imaging. She underwent multiple bilateral AVM embolization 17 years ago. She had minimal shortness of breath without epistaxis, hemoptysis, hematemesis or hematochezia. One month ago, her chest X-ray revealed multiple embolization coils bilaterally (A). Computed tomography (CT) chest scan without contrast showed multiple embolization coils, multiple tortuous vascular malformations in both lungs with enlargement of left lower lobe AVM (B). Magnetic resonance imaging (MRI) brain 17 years ago did not show brain vascular malformation. The diagnosis of progressive Osler-Weber-Rendu or hereditary hemorrhagic telangiectasia (HHT) was made. She was referred to HHT center and underwent coiling. After that, she has been clinically stable and a repeat CT chest on the follow-up visit showed smaller left lower lobe AVM (B), so conservative management was planned.

**Figure 1:**
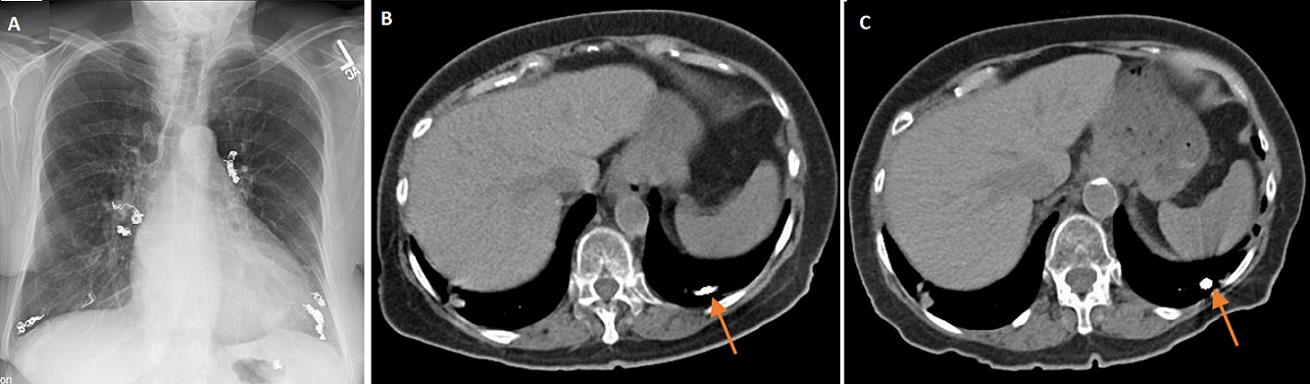
A) chest X-ray showing multiple embolization coils bilaterally; B) CT chest without contrast showing large AVM at left lower lobe (arrow); C) a repeat CT chest without contrast one year later showing smaller AVM at left lower lobe after coiling (arrow)

